# Isolation and Characterization of a Bacterial Strain Capable of Efficient Berberine Degradation

**DOI:** 10.3390/ijerph16040646

**Published:** 2019-02-21

**Authors:** Shiyue Liu, Yi Zhang, Ping Zeng, Heli Wang, Yonghui Song, Juan Li

**Affiliations:** 1 School of Water Resources and Environment, China University of Geosciences (Beijing), 20 Chengfu Road, Haidian District, Beijing 100083, China; liushiyue5@126.com (S.L.); wangheli@cugb.edu.cn (H.W.); 2 Chinese Research Academy of Environmental Sciences, 8 Dayangfang, Chaoyang District, Beijing 100012, China; songyh@craes.org.cn (Y.S.); lijuan@craes.org.cn (J.L.); 3 Fudan University, 2005 Songhu Road, Yangpu District, Shanghai 200438, China; zhang-yi@fudan.edu.cn

**Keywords:** Berberine, *Sphingopyxis* sp., 16S rDNA, degradation kinetics

## Abstract

*Background:* Berberine (BBR) is a pharmaceutical chemical with a broad antibacterial spectrum, and its biological treatment has been of research and practical interest. In this study, a pure bacterial strain B16 was isolated from the activated sludge in a pharmaceutical wastewater treatment plant. The aim of the study is to characterize the properties of the strain B16, especially its BBR degradation capability. *Methods:* The identification of strain B16 was conducted by visual observation, as well as biochemical and phylogenetic analysis. The degradation kinetics of strain B16 was tentatively described by Haldane model. *Results:* The strain B16 was 100% determined as a *Sphingopyxis* sp. The kinetic parameters of BBR degradation by strain B16 were as follows: Vmax 54.73 ± 5.54 mg (g MLSS · h)^−1^, Km 66.68 ± 8.95 mg L^−1^, and Ki 43.16 ± 5.92 mg L^−1^, with an R^2^ of 0.996. Stain B16 exhibited considerable capability of BBR degradation. BBR of initial concentration 40 mg L^−1^ could be completely degraded in 48 h under optimal conditions. *Conclusions:* strain B16 was the first pure culture found with the ability to totally mineralize BBR, indicating the potential of B16 application in real industrial processes.

## 1. Introduction

Berberine (5,6−Dihydro−9,10−dimethoxybenzo [g]−1,3−benzodioxolo [5,6−α] quinolizinium, C_20_H_18_NO_4_, BBR in short) is an isoquinoline quaternary alkaloid isolated from many kinds of medicinal plants, such as *Berberis aristata*, *Berberis aquifolium*, *Berberis vulgaris*, *Coptis chinensis*, *Coptis japonica*, *Hydrastis canadensis*, *Phellodendron amurense,* and *Phellodendron chinense schneid*. BBR can be extracted from herbal plants or synthesized by chemical means, and applied as a natural antibiotic against various bacteria [1]. Due to its anticancer, anti−inflammatory and antibiotic properties, the application of BBR has been expanded to antitumor, anti−oxidation, anti−Alzheimer’s disease, and anti−hyperglycemic applications [2,3], resulting in a sharp increase in demand for BBR. Meanwhile, the extensive production and use of BBR lead to the discharge of large quantity of BBR−containing wastewaters into the environment, posing a great threat to ecosystems because of its substantial inhibitory effects on biological activities [4]. Therefore, the treatment of BBR in wastewaters, especially those from pharmaceutical processes, is necessary before its discharge into the environment.

The common treatment methods of BBR wastewaters include physical, chemical and biological processes. The physical−chemical treatments of BBR wastewaters have limited application due to their high cost and risk of producing new pollutants [4,5]. Biological treatment is therefore preferred because of its lower cost and potential of complete mineralization. Degradation of BBR in wastewaters by a mixed microbial culture has been reported before [6]. It was reported that after 118 days of operation, 99.0%, 98.0% and 98.0% overall removals of BBR, COD and NH_3_ −N were achieved, respectively. The mixed microbial culture was dominated by *Firmicutes*, *Bacteroidetes*, *Proteobacteria*, Alpha− and Beta−*proteobacteria*, *Hydrogenophaga*, *Azoarcus*, *Sphingopyxis*, *Stenotrophomonas*, *Shinella* and *Alcaligenes*. Aerobic granular sludge has also been successfully cultivated with the effluent from an anaerobic baffled reactor which degraded BBR wastewater [7]. In this study, the microbial morphology, population, structure succession, community diversity and dominant bacteria species were investigated. The results indicated that many types of dominant bacteria existed in the aerobic granular sludge [7]. Furthermore, some individual microbial strains have also been found to have the ability to aerobically degrade BBR. Takeda et al. isolated BBR−utilizing bacteria BD3100 from soil, which was identified as a *Sphingobium* sp. BD3100 was able to convert BBR into 11−hydroxyberberine, an intermediate of BBR degradation, in 6 days [8]. The research on biological treatment of BBR, especially in pharmaceutical wastewaters, is still very limited, and few pure cultures have been identified to possess the ability to degrade high strength BBR. To further investigate the enhancement of BBR biological treatment, it is of great significance to explore the properties of microorganisms capable of BBR degradation. Therefore, in this study, a bacterium B16 with BBR−degrading ability was isolated from the activated sludge of a pharmaceutical wastewater treatment plant, and its genetic and physiological characteristics were investigated in detail. Its phylogeny and morphology were characterized by 16S rDNA−based methods and electron microscopy, respectively. The BBR degradation capability of strain B16 was further studied, which could provide vital information for its further application in real treatment processes, e.g., through bio−augmentation.

## 2. Materials and Methods 

### 2.1. Enrichment and Isolation of a BBR−Degrading Culture

#### 2.1.1. Source of Inoculation

The seed sludge for isolation was collected in a pharmaceutical wastewater treatment plant with an influent containing BBR from the production unit, containing BBR concentrations estimated to be 1 to 5 mg/L. The main processes of this plant consisted of a hydrolysis−acidification unit followed by a contact oxidation process. The sludge was collected from the contact oxidation tank, and the samples were placed in sterile polyethylene bottles, transported to the laboratory, and then maintained at 4 °C until use.

#### 2.1.2. Isolation of Pure Cultures and Initial Screening

For screening and cultivation of BBR−utilizing bacteria, a mineral salt medium (MSM) was used, with the composition of: (NH_4_)_2_SO_4_ 1 g L^−1^, KH_2_PO_4_ 0.8 g L^−1^, K_2_HPO_4_ 0.2 g L^−1^, MgSO_4_ · 7H_2_O 0.5 g L^−1^, FeSO_4_ 10 mg L^−1^, CaCl_2_ 50 mg L^−1^, NiSO_4_ 32 mg L^−1^, Na_2_B_4_O_7_ · 10H_2_O 7.2 mg L^−1^, (NH_4_)_6_Mo_7_O_2_ · 4H_2_O 14.4 mg L^−1^, ZnCl_2_ 23 mg L^−1^, CoCl_2_ · 6H_2_O 21 mg L^−1^, CuCl_2_ · 2H_2_O 10 mg L^−1^ and MnCl_2_ · 4H_2_O 30 mg L^−1^. The initial pH of MSM was adjusted to 7.0, and BBR (30.0 mg L^−1^) was supplemented as the sole source of carbon and energy. All the medium ingredients were aseptically prepared.

About 0.1 g (wet weight) of activated sludge was aseptically mixed with 20 mL of MSM in a sterilized beaker to separate the cells. The mixture was centrifuged at 3000 rpm (Eppendorf 5430, Germany) for 5 min. The supernatant was diluted with MSM to 10^−1^ to 10^−7^ fold. 100 µL of each dilution was then spread onto an agar plate, made up with 10 mg L^−1^ BBR and 2.5% (W/V) agar in MSM (Shanghai Sinopharm, China). The plates were inverted and cultivated at 30 °C, and the growth of colonies was monitored. When a single colony appeared, it was transferred to a fresh plate, and the incubation process was repeated until pure cultures were obtained. Through microscopic observation, the purity of the cultures was primarily confirmed, and the strains were stored in glycerol at −80 °C.

At the end of this stage, 18 pure cultures were obtained. They were subsequently screened based on their ability to grow of BBR. Growth experiments were performed in 200 mL MSM solutions with 20 mg L^−1^ of BBR. The growth rate and specific degradation rate exhibited by the individual strains were compared and used as the criteria of screening, and one strain, B16 was chosen for further and more detailed study because of its fastest growth.

### 2.2. Characterization of the Isolated Strain

#### 2.2.1. Colony and Cell Morphology

Colony morphology was assessed by visual observation of its growth on solid medium under microscope. Cells were obtained from fully developed colonies, and Gram−staining was performed. Cellular morphology has observed by light microscopy (Leica M5100, Wetzlar, Germany) and SEM (SU−70, Hitachi, Ibaraki, Japan).

#### 2.2.2. Identification of Biochemical Characteristics

The biochemical profile of the isolate was determined with API 20NE strips (bioMérieux SA, Craponne, France) following the manufacturer’s instructions [9]. The identification was performed using the APILAB Plus database (bioMérieux SA, Craponne, France) [10].

#### 2.2.3. DNA Extraction, PCR, Sequencing and Phylogenetic Analysis

16S rDNA genetic analysis of the isolate was performed by Sangon Biotech Co., Ltd. (Shanghai, China). The total genomic DNA extraction was performed using the Ezup Genomic DNA Extraction Kit SK8255 (Sangon Biotech Co., Ltd. Shanghai, China) following the manufacturer’s protocol. The purified DNA amplicons were sequenced using a DNA Sequencer (Applied Biosystems 3730−XL, Waltham, Massachusetts, USA) at Sangon Biotech Co., Ltd. (Shanghai, China) according to standard protocols.

Homology searching was performed using GenBank DNA databases (National Center for Biotechnology Information, Rockville Pike, Bethesda MD, USA) by the BLAST program (U.S. National Library of Medicine, 8600 Rockville Pike, Bethesda, USA) [11]. The sequence from strain B16 was used for the calculation of phylogenetic analysis. Nucleotide substitution rates were determined by CLUSTAL W [12]. The neighbor−joining method was used to reconstruct a phylogenetic tree from the distance matrices, where the percentage of replicate trees in which the associated taxa clustered together in the bootstrap test (1000 replicates) are shown next to the branches [13]. The tree was drawn to scale, with branch lengths in the same units as those of the evolutionary distances used to infer the phylogenetic tree. The evolutionary distances were computed using the Maximum Composite Likelihood method [14] and are in the units of the number of base substitutions per site. Evolutionary analyses were conducted in MEGA7 [15].

### 2.3. Tests on Degradation Characteristics

#### 2.3.1. Operational Conditions

Experiments were performed in triplicate in 500 mL shake flasks. Each flask contained 200 mL of MSM with 20 mg L^−1^ of BBR. The pH value of the medium was adjusted with NaHCO_3_. Sterile controls were prepared by autoclave at 121 °C for 20 min before the introduction of BBR.

The strain B16 was introduced to give the culture an optical density (OD_600_) around 0.3, and the culture medium was incubated at pH 7, 25 °C and 150 rpm. These conditions were set as the standard operational parameters of the test. The concentration of BBR was measured at regular intervals during the incubation to trace its removal. The operational conditions tested included the inoculation volume ratio (1%, 5%, 10%, 15% and 20%), initial pH (5, 6, 7, 8 and 9), incubation temperature (20 °C, 25 °C, 30 °C and 35 °C), and rotational speed (90 rpm, 120 rpm, 150 rpm and 180 rpm). When one condition was being tested, the remaining three parameters were set at the standard values. The optimal conditions were determined based on the removal efficiency of BBR.

#### 2.3.2. Growth Characterization of the Isolated Strain

The strain was inoculated into and incubated in 250 mL flasks containing MSM and BBR. The initial BBR concentrations were set at 20 mg L^−1^, 40 mg L^−1^, 60 mg L^−1^, 80 mg L^−1^ and 100 mg L^−1^ respectively, and the initial concentration of the strain was set at 0.1 g L^−1^ (dry weight). For all experiments, samples were inoculated and incubated at 25 °C and 150 rpm, and each test was performed in triplicate. The growth of the culture was determined by measuring the biomass abundance in the mixture.

#### 2.3.3. Kinetic Model

Experiments were performed in MSM with the initial BBR concentration ranging from 0 to 100 mg L^−1^ under the optimal conditions. Haldane model was adopted to predict the biodegradation kinetics of inhibitory substrates. First, zero−order reaction kinetics was used to fit the rate of BBR disappearance in the initial minutes of reaction, and the specific degradation rate was calculated under each initial BBR concentration [16]. The specific rates were then plotted against the BBR concentrations, and the fitting of Haldane kinetics was done with ORIGIN 2018 software (OriginLab Corporation, Northampton, Massachusetts, USA).

### 2.4. Analytical Methods

#### 2.4.1. BBR

BBR concentrations were determined by high−pressure liquid chromatography (HPLC, Agilent 1260 Infinity, Santa Clara, California, USA). Liquid samples were filtered through 0.2−µm−pore−size PVDF Acrodisc Minispike syringe filters (Pall Gelman Laboratory, Ann Arbor, Michigan) before analysis. 20 μL BBR samples were injected into a HB−C8 column (150 × 4.6 mm, 5 μm) maintained at 30 °C. Acetonitrile / KH_3_PO_4_ (0.02 mol L^−1^) (30/70, *v*/*v*) was chosen as the mobile phase at an applied flow rate of 1.0 mL min^−1^. The separated components were detected by a UV detector (Spectro flow 773, Spectronics Coperation, Westbury, New York, USA) at a wavelength of 345 nm. Each measurement was performed in triplicate [17].

#### 2.4.2. Biomass Abundance

The abundance of the culture was determined by measuring its dry weight after filtration.

#### 2.4.3. TOC

Aqueous TOC was tested with a total organic carbon analyzer (SHIMADZU TOC−L, Kyoto, Japan).

## 3. Results

### 3.1. Morphology and Physical Characteristics of Strain B16

**Figure 1:**
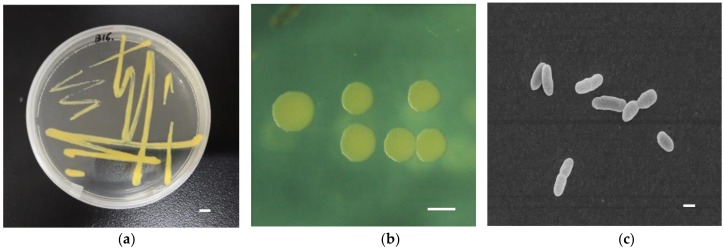
Pictures of B16: (**a**) B16 colonies on agar (bar: 4 mm); (**b**) B16 colonies on agar (bar: 1 mm) and (**c**) scanning electron images of B16 cells (bar: 4 µm).

The morphology of strain B16 is shown in Figure 1. B16 is a Gram−negative, non−sporulating and rod−shaped bacterium. Cells were between 5.0 µm and 9.0 µm in length, and 0.2 µm–0.4 µm in diameter when grown in BBR−MSM media at 30 °C. The B16 colonies formed on BBR−MSM agar plates were yellow, convex and circular, and had a smooth surface. B16 did not possess auto−aggregation abilities.

### 3.2. Biochemical Properties of Strain B16

The biochemical properties of strain B16 were determined by API 20NE strips. The results of potassium nitrate (NO3), tryptophan (TRP), glucose (GLU), arginine (ADH), gelatin (GEL), mannitol (MAN), gluconate (GNT), capric acid (CAP), adipic acid (ADI), malic acid (MLT), citric acid (CIT) and phenylacetic acid (PAC) were negative, while the results of urea (URE), esculin (ESC), p−nitro−β−D methyl galactose (PNPG), glucose (GLU) second, arabinose (ARA), Mannose (MNE), N−acetyl−glucosamine (NAG), maltose (MAL) and tetramethyl−p− phenylenediamine (OX) were positive.

The APILAB PLUS database was used for further analysis of API 20NE results. The result for B16 was 0,663,304, which showed that strain B16 belonged to *Sphingopyxis* sp., with an identification index of 91.7%, and a taxonomy index T of 0.74. 

### 3.3. Microbial Identification and Phylogenetic Analysis

**Figure 2:**
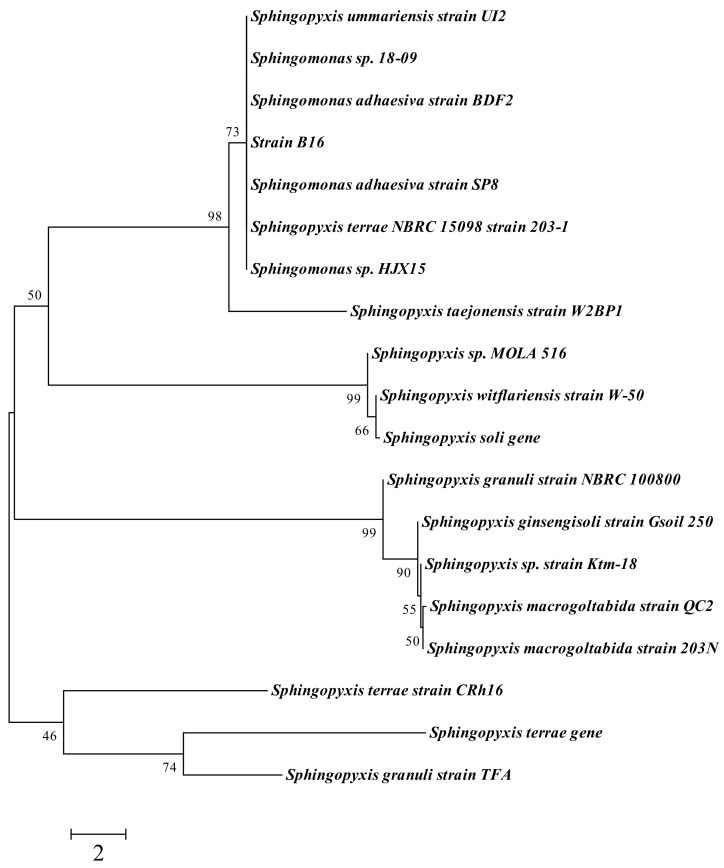
16S rDNA phylogenetic tree for strain B16 calculated by the neighbor joining method. The numbers at the branch nodes are bootstrap values based on 100 resamplings. Only bootstrap values greater than 50% are shown. Scale bar indicates Nucleotide divergence of 1%. Nucleotide sequence accession number for each reference strain is indicated in brackets.

Phylogenetic analysis based on 1258 unambiguous bases revealed that strain B16 was a member of the *Sphingopyxis* genus. The gene sequences were submitted to CICC (WDCM582) and NCBI, and the accession number of B16 in CICC (WDCM582) is CICC 24640, while the accession number in GenBank is MH301295. Figure 2 illustrates the phylogenetic relationship of strain B16 with species of close similarity, among which *Sphingopyxis terrae* NBRC 15098 strain 203−1 is the closest, with 100% similarity (Table 1).

### 3.4. Degradation of Organic Substances by Strain B16

In this study, 27 organic compounds (raw materials or intermediate products of medicines) with different chemical properties were chosen to test the metabolic capability of strain B16. Incubation was conducted on agar plates containing these chemicals as the sole organic carbon sources (all at the initial concentration of 20 mg L^−1^) at 25 °C for 48–72 h, and the growth of colonies was observed with time. As shown in Table 2, among the 27 tested chemicals, 5 could not support the growth of strain B16, including formaldehyde, vanillin, dimethyl sulfate, methyl dichloroacetate and p−methylphenol. The other 22, including benign substances such as sodium acetate, urea, acetic acid, methanol, ethanol; and toxic chemicals such as phenol, 3,5−dimethylphenol, and chloramphenicol; and refractory chemicals such as benzene, dimethyl phthalate, di−n−butyl phthalate, and diisobutyl phthalate, could support the growth of strain B16. The results indicated that strain B16 could utilize chemicals with different properties as the sole carbon and energy source besides BBR, demonstrating its versatility and potential in real applications. More notably, B16 showed tolerance to multiple toxic compounds, and the ability to cleave aromatic rings in their structures, which might contribute to its capability of BBR degradation.

### 3.5. Optimization of Growth Conditions

**Figure 3:**
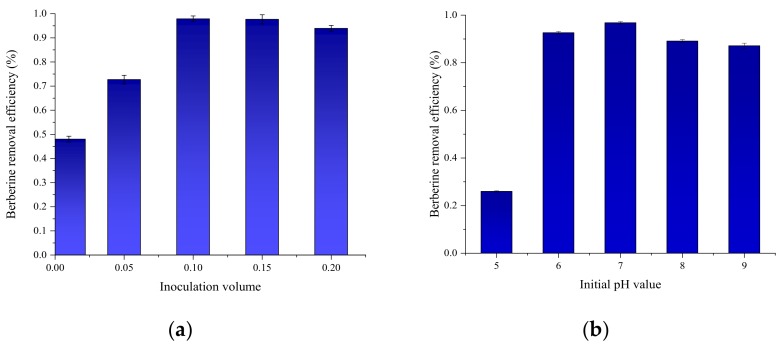

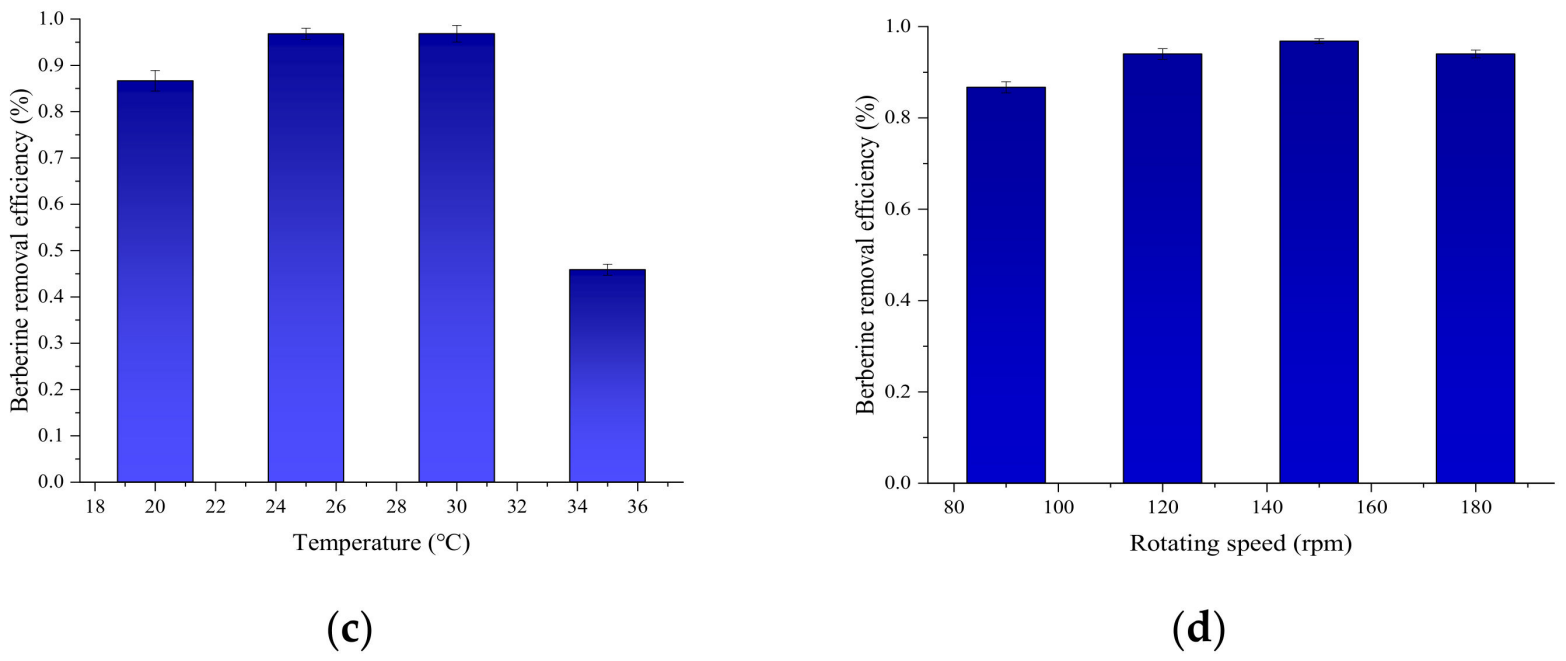
The influence of different parameters: (**a**) Inoculation volume; (**b**) Initial pH value; (**c**) Temperature and (**d**) Rotating speed.

Several common operational conditions were first studied for their effects on B16 growth, which include inoculation ratio, pH, temperature and mixing strength. Their influences on BBR removal efficiency are depicted in appendix. When the inoculation ratio of strain B16 increased from 1% to 10% (*v*/*v*), BBR removal efficiency increased from 48% to 98.5%, which showed little improvement at the higher ratio of 15%. Then, BBR removal decreased to 94% at the inoculation volume of 20%. As to the initial pH, BBR removal efficiency increased from 26% to 93% when pH level increased from 5 to 6, then rose to 97% when pH reached 7. BBR removal rate began to decline with further rise of pH, i.e., 89% at pH 8, and finally 87% at pH. In terms of temperature, BBR removal efficiency increased from 87% to 97.5% when temperature increased from 20 °C to 25 °C, then decreased to 96.5% at 30 °C. Finally, it decreased to 47% when the temperature reached 35 °C. The mixing in the test was provided by a rotary shaker, and rotational speed was also tested as an operation condition. BBR removal efficiency increased from 87% to 94% when the speed increased from 90 rpm to 120 rpm, then reached the highest of 97.8% at 150 rpm. However, the removal decreased to 93% when rotational speed reached 180 rpm. Thus, it can be inferred that the optimal operational conditions for B16 were as follows: inoculation ratio range 10–15%, pH range 6–7, temperature range 25–30 °C, and rotational speed 150 rpm (Figure 3).

### 3.6. The Growth of Strain B16 and Degradation Kinetics of BBR

**Figure 4:**
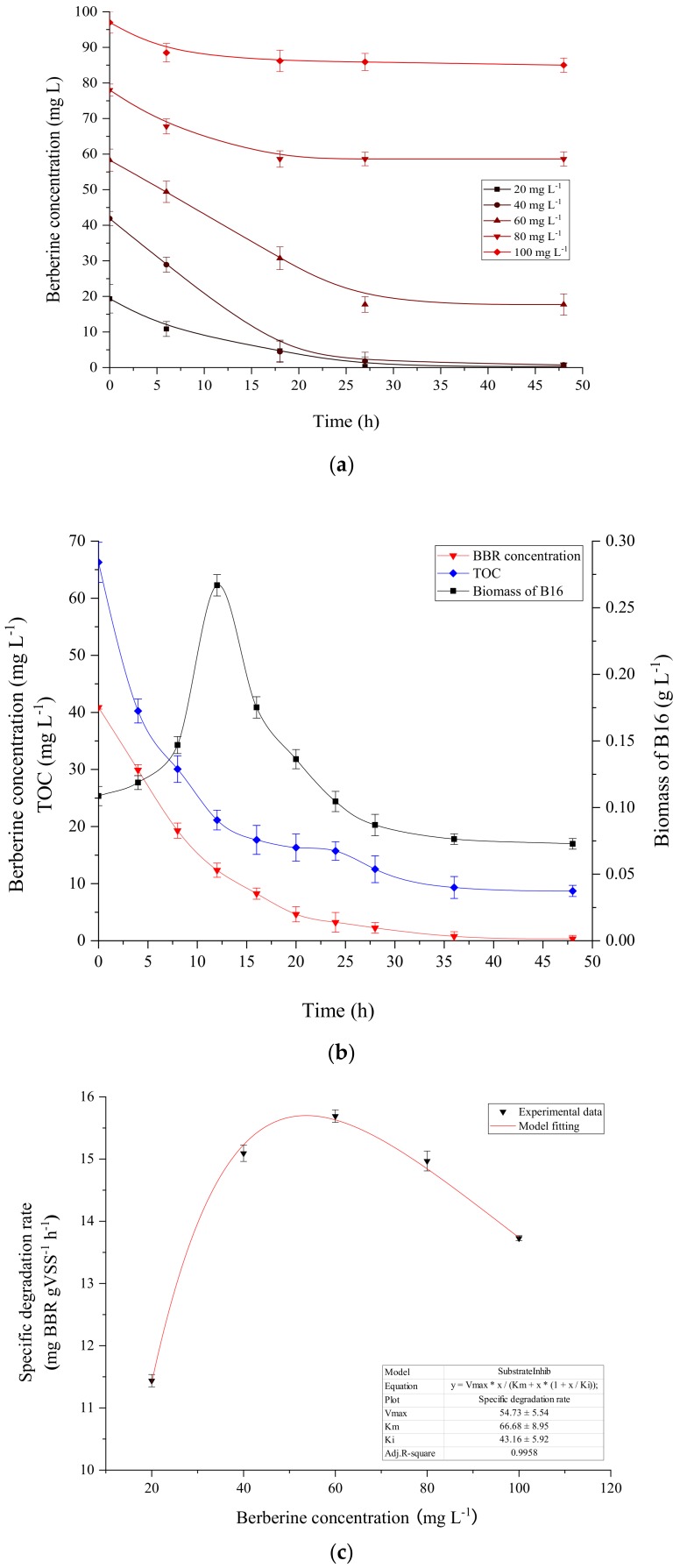
(**a**) Degradation of BBR at different concentrations by strain B16; (**b**) The growth of strain B16 and degradation of BBR at the initial concentration of 40 mg L^−1^, (**c**) Specific degradation rates of strain B16 at different BBR concentrations.

Some hydrocarbon contaminants are known to have inhibitory effect on the activity of biomass [18], the degree of which is correlated to the concentrations of the inhibitors. Because of the antibiotic property of BBR, its initial concentration might be expected to have an inhibitory effect on the biodegradation process. To evaluate the degradation of BBR by strain B16, batch experiments were carried out at different initial BBR concentrations ranging from 0 mg L^−1^ to 100 mg L^−1^. Figure 4a showed the results of the effect of initial BBR concentrations on the biodegradation process at pH 7 and 25 °C. 

The results show various degrees of BBR degradation at different initial concentrations within 48 h. BBR could be completely degraded at concentrations of 20 mg L^−1^ and 40 mg L^−1^. The degradation efficiency decreased to 69.61% when the concentration was 60 mg L^−1^. When the concentration rose to 80 mg L^−1^, the degradation efficiency dropped to 24.83%. When the concentration of BBR reached 100 mg L^−1^, the degradation efficiency was only 12.37% after 48h incubation. This indicated that BBR had a considerable inhibitory effect on the biodegradation capabilities of B16, and the inhibition was positively correlated to its initial concentration.

To further investigate the relationship between the growth of strain B16 and degradation of BBR, a batch experiment was carried out at the initial BBR concentration of 40 mg L^−1^ at pH 7.0 and 25 °C. The results are shown in Figure 4b. The initial biomass abundance of strain B16 was 0.1 g L^−1^, and after 12 h of continuous reaction, the biomass reached a maximum of 0.27 g L^−1^. Then, it decreased slowly and remained stable after dropping to 0.08 at around 36 h, at which point BBR was completely removed. The change of BBR concentration correlated negatively with biomass variation in the graph, indicating that this was a growth−related degradation. Figure 4b also displays the change of TOC with reaction time, and it showed a parallel trend to BBR, indicating concomitant mineralization of BBR. After 36 h of continuous incubation, TOC decreased to around 10 mg L^−1^ and remained stable for the rest of the test time, confirming that BBR supported B16 growth as the sole organic carbon and energy source. In order to explore the dependence of degradation rate on BBR concentration, the degradation kinetics of BBR was investigated under the optimal conditions.

The experimental results of BBR degradation rates under different initial concentrations were calculated. Strain B16 was capable of degrading BBR at concentrations up to 100 mg L^−1^. Based on the data obtained, Haldane kinetic parameters were estimated through nonlinear fitting [19]. Predicted degradation rates of strain B16 were compared with the experimental data, and the results are shown in Figure 4c. The Haldane parameters for strain B16’s degradation of BBR were obtained, with V_max_ of 54.73 ± 5.54 mg (g MLSS · h)^−1^, Km of 66.68 ± 8.95 mg L^−1^ and Ki of 43.16 ± 5.92 mg L^−1^ (R^2^ = 0.9958).

According to the model’s prediction, degradation rates increase with increasing initial BBR concentrations, and can reach an actual maximum specific rate of 15.70 mg BBR (g MLSS · h)^−1^ at the concentration of 53.63 mg L^−1^. Beyond this value the removal rate decreases, indicating the inhibitory effect of BBR on strain B16.

## 4. Discussion

BBR, a quaternary ammonium salt, is a major component of the protoberberine group of benzylisoquinoline alkaloids found in such plants as *Berberis*, and considered to be a type of antibiotic. The structure of BBR is displayed in the appendix, and its anti−inflammatory properties were found to be related with the quaternary ammonium on number C benzene ring [20]. Microbial degradation of BBR was often hindered by toxicity effects exerted at high concentrations, which may harm microbial cells by damaging the cytoplasmic membrane and deactivating enzymes [21,22]. 

For different microorganisms, BBR showed different inhibition activities. Wei et al. [23] found that BBR inhibited the growth of various species in genus *Candida*, including *C. albicans*, *C. glabrata*, *C. kefyr*, *C. krusei*, *C. parapsilosis*, and *C. tropicalis*, at the minimum inhibitory concentrations (MIC) of 0.98–31.25 mg L^−1^. In addition, BBR exhibited synergistic effects with commonly used antimycotic drugs against *C. albicans*, either in planktonic or in biofilm growth phases. Li et al [2]. discovered that MICs of BBR against resistant *E. coli*, *B.subtilis* and *S. cerevisiae* were 270 μg mL^−1^, 210 μg mL^−1^ and 120 μg mL^−1^, respectively. Thus, these resistant bacterial strains showed higher tolerance than *C. albicans*. Yan [24] studied the inhibitory effects of BBR alkaloids on *Bifidobacterium adolescentis*. The results showed that the half inhibition concentration (IC50) of BBR on *Bifidobacterium adolescentis* was 790.3 μg mL^−1^. It can be seen that BBR has a strong inhibitory effect on many microorganisms, and it is not easy to find a microorganism which could tolerate high concentrations of BBR, even in biological wastewater treatment systems. 

Some microorganisms with BBR degradation ability were reported in previous studies. Qiu [16] cultivated aerobic granular sludge to degrade BBR in a membrane bioreactor (MBR) with BBR and COD removal efficiency of 99% and 98%, respectively. The mixed cultures, including *Hydrogenophaga*, *Azoarcus*, *Sphingomonas* (which was later reclassified in the genus *Sphingopyxis*) [25], *Stenotrophomonas*, *Shinella*, *Alcaligenes*, and *Nitrospir* in the aerobic granules, were identified as the functional species in the biodegradation of BBR and/or its metabolites [6]. BBR was also reported to be removed by mixed cultures in soil. The highest concentration of BBR under which the soil bacteria displayed removal activity was 90 mg L^−1^, and the BBR was completely removed in 12 days. Three pure cultures were then isolated from the soil bacteria consortium, and identified as *Bacillus* sp. They were found to have difficulty surviving when BBR was provided as the sole carbon source [2].

In this study, a strain B16 with BBR degradation capability was isolated from the activated sludge in a pharmaceutical wastewater treatment plant, which was identified as a *Sphingopyxis* sp. This strain demonstrated considerable capability of BBR removal up to 60 mg L^−1^ initial BBR concentration. In addition, BBR of concentrations 20 mg L^−1^ and 40 mg L^−1^ could be completely mineralized within 48 h. To the authors’ knowledge, this is the first time that a pure strain is reported to have the ability to completely degrade and mineralize BBR. The kinetics and resistance exhibited towards BBR were tentatively described by Haldane model. 

The Haldane model is a type of biological inhibition model. It was chosen to describe microbial degradation kinetics of BBR because of its mathematical simplicity and wide utilization in modeling the degradation kinetics of inhibitory substrates. 

The fitting results showed that strain B16 could tolerant BBR concentration higher than 100 mg L^−1^. The parameters to describe BBR degradation kinetics by strain B16 were: V_max_ of 54.73 ± 5.54 mg (g MLSS · h)^−1^, Km of 66.68 ± 8.95 mg L^−1^, Ki of 43.16 ± 5.92 mg L^−1^. In comparison, the mixed culture of aerobic granules in an AGMBR showed an apparent V_max_ of 120.8 mg (g MLSS · h)^−1^, Km of 404.3 mg L^−1^ and Ki of 97.8 mg L^−1^ [16]. Therefore, the V_max_, K_m_ and K_i_ values exhibited by this pure culture were lower than those of the mixed culture aerobic granules. As the tests were performed by different methods on different cultures, their results are not directly comparable. However, these parameters might offer a useful indication as to the ability and potential of B16 in real applications. K_m_ of B16 was much lower than the granules, which suggested a much higher affinity to BBR, and high activity at low concentrations. On the other hand, B16’s lower K_i_ indicates higher inhibition by BBR on this pure culture, so did its lower V_max_. This is understandable as aerobic granules are microbial aggregates, whose structure could offer protection against toxic substances [26]. However, B16 exhibited the ability of total BBR removal (40 mg L^−1^) within 48 h, while it took the mixed culture of soil bacteria 7 days to completely remove BBR [8]. This showed the potential of B16 application in real applications. 

In addition, *Sphingopyxis,* the genus B16 belongs to, was identified in BBR degradation aerobic granules, indicating that it was one of the functional species for BBR degradation [6]. Furthermore, *Sphingopyxis* was found to possess the capability of cleaving nitrogen heterocyclic ring and aromatic rings, which are related to antibiotic resistance [27]. As B16 was only one of the isolates from the consortium, it is possible that various ecological niches existed in the mixed culture, where different microbes worked in concert to degrade BBR efficiently. The exploration on the ecological diversity and functions of various bacteria strains in the consortium is under way.

## 5. Conclusions

In this study, a bacterial strain B16 with BBR degradation capability was isolated from the activated sludge in a pharmaceutical wastewater treatment plant. The strain was 100% identified as a *Sphingopyxis* sp, one of the reported functional species for BBR degradation. B16 exhibited a considerable capability of BBR degradation, and the degradation kinetics was tentatively described by Haldane model. The parameters were as follows: Vmax 54.73 ± 5.54 mg (g MLSS · h)^−1^, Km 66.68 ± 8.95 mg L^−1^, and Ki 43.16 ± 5.92 mg L^−1^, with an R^2^ of 0.996. In addition, strain B16 was found to be the first pure culture with the ability to totally mineralize BBR. Under the optimal conditions of inoculation ratio 10%, pH 7, temperature 25 °C, and rotational speed 150 rpm, BBR with initial concentration of 40 mg L^−1^ could be completely degraded in 48 h, indicating the potential of B16 application in real industrial processes.

## Figures and Tables

**Table 1:** Comparative result of gene sequence with NCBI.

Accession	Description	Max Score	Total Score	Query Coverage	Evalue	Max Ident
CP013342.1	*Sphingopyxis terrae*; NBRC 15098	2318	2318	100%	0.0	99%
KP979546.1	*Sphingopyxis* sp.; HJX15	2318	2318	100%	0.0	99%
AB680906.1	*Sphingopyxis macrogoltabida*; NBRC 15593;	2318	2318	100%	0.0	99%
MK217492.1	*Sphingopyxis* sp.; YC−JH3	2318	2318	100%	0.0	99%

**Table 2:** The growth of B16 on agar plate with different substrates.

Substrate	Growth	Substrate	Growth
Benzene	+	Tetrabutylammonium bromide	++
Chlorobenzene	+	Sodium acetate	++
Catechol	+	Urea	++
Formaldehyde	−	Phenol	+
Benzaldehyde	++	P−methylphenol	−
Glyoxal	+	3,5−dimethylphenol	++
Vanillin	−	Acetophenone	+
Acetic acid	+	Benzothiazole	++
Methanol	++	Benzyl benzoate	++
Ethanol	+	Dimethyl phthalate	++
Sorbitol	++	Di−n−butyl phthalate	++
Isopropanol	++	Diisobutyl phthalate	++
Dimethyl sulfate	−	Chloramphenicol	+
Methyl dichloroacetate	−		

“+” indicates the growth status of B16.

## References

[1] Wojtyczka R.D., Arkadiusz D., MalGorzata K., Robert K., Agata K.-D., Tomasz M., Danuta I. (2014). Berberine enhances the antibacterial activity of selected antibiotics against coagulase−negative *Staphylococcus* strains in vitro. Molecules.

[2] Li Y., Yin Y.M., Wang X.Y., Wu H., Ge X.Z. (2017). Evaluation of berberine as a natural fungicide: Biodegradation and antimicrobial mechanism. J. Asian Nat. Prod. Res..

[3] Othman M.S., Safwat G., Aboulkhair M., Abdel Moneim A.E. (2014). The potential effect of berberine in mercury−induced hepatorenal toxicity in albino rats. Food Chem. Toxicol..

[4] Qin W., Song Y., Dai Y., Qiu G., Ren M., Zeng P. (2015). Treatment of berberine hydrochloride pharmaceutical wastewater by O_3_/UV/H_2_O_2_ advanced oxidation process. Environ. Earth Sci..

[5] Ren M., Song Y., Xiao S., Ping Z., Peng J. (2011). Treatment of berberine hydrochloride wastewater by using pulse electro−coagulation process with Fe electrode. Chem. Eng. J..

[6] Qiu G., Song Y.H., Zeng P., Duan L., Xiao S. (2013). Characterization of bacterial communities in hybrid upflow anaerobic sludge blanket (UASB)−membrane bioreactor (MBR) process for berberine antibiotic wastewater treatment. Bioresour. Technol..

[7] Liu F., Song Y., Zeng P., Wang Q., Han S., Song B., Lin H.U. (2014). Cultivation of Aerobic Granules with Effluent from ABR and Analysis of Their Microbial Diversity of Microbial Communities. Environ. Sci. Technol. (Chin.).

[8] Takeda H., Ishikawa K., Wakana D., Fukuda M., Sato F., Hosoe T. (2015). 11−Hydroxylation of Protoberberine by the Novel Berberine−Utilizing Aerobic Bacterium *Sphingobium* sp. Strain BD3100. J. Nat. Prod..

[9] Guillaumegentil O., Sonnard V., Kandhai M.C., Marugg J.D., Joosten H. (2005). A simple and rapid cultural method for detection of *Enterobacter sakazakii* in environmental samples. J. Food Prot..

[10] Rojas I., Bernardi L., Ratsch E., Kania R., Wittig U., Saric J. (2002). A database system for the analysis of biochemical pathways. Silico Biol..

[11] Altschul S.F., Madden T.L., Schaffer A.A., Zhang J., Zhang Z., Miller W., Lipman D.J. (1997). Gapped BLAST and PSI−BLAST: A new generation of protein database search programs. Nucleic Acids Res..

[12] Thompson J.D., Higgins D.G., Gibson T.J. (1994). CLUSTAL W: Improving the sensitivity of progressive multiple sequence alignment through sequence weighting, position−specific gap penalties and weight matrix choice. Nucleic Acids Res..

[13] Saitou N., Nei M. (1987). The neighbor−joining method: A new method for reconstructing phylogenetic trees. Mol. Biol. Evol..

[14] Tamura K., Nei M., Kumar S. (2004). Prospects for inferring very large phylogenies by using the neighbor−joining method. Proc. Natl. Acad. Sci. USA.

[15] Kumar S., Stecher G., Tamura K. (2016). MEGA7: Molecular Evolutionary Genetics Analysis Version 7.0 for Bigger Datasets. Mol. Biol. Evol..

[16] Qiu G. (2011). Hybrid UASB−MBR Process for Berberine Pharmaceutical Wastewater Treatment and the Process Mechanisms. Ph.D. Thesis.

[17] Yi L., Xu X. (2004). Study on the precipitation reaction between baicalin and berberine by HPLC. J. Chromatogr. B.

[18] Ruan B., Wu P., Chen M., Lai X., Chen L., Yu L., Gong B., Kang C., Dang Z., Shi Z. (2018). Immobilization of *Sphingomonas* sp. GY2B in polyvinyl alcohol–alginate–kaolin beads for efficient degradation of phenol against unfavorable environmental factors. Ecotoxicol. Environ. Saf..

[19] Zeng P., Moy B., Song Y., Tay J. (2008). Biodegradation of dimethyl phthalate by *Sphingomonas* sp. isolated from phthalic−acid−degrading aerobic granules. Appl. Microbiol. Biotechnol..

[20] Iwasa K., Kamigauchi M., Sugiura M., Nanba H. (1997). Antimicrobial activity of some 13−alkyl substituted protoberberinium salts. Planta Med..

[21] Jin J.L., Hua G.Q., Meng Z. (2011). Antibacterial Mechanisms of Berberine and Reasons for Little Resistance of Bacteria. Chin. Herb. Med..

[22] Mohsen I., Hossein H. (2010). Pharmacological and therapeutic effects of Berberis vulgaris and its active constituent, berberine. Phytother. Res..

[23] Wei G.X., Xu X., Wu C.D. (2011). In vitro synergism between berberine and miconazole against planktonic and biofilm *Candida* cultures. Arch. Oral Biol..

[24] Yan D., Han Y.M., Wei L., Xiao X.H. (2009). Effect of berberine alkaloids on *Bifidobacterium adolescentis* growth by microcalorimetry. J. Therm. Anal. Calorim..

[25] Takeuchi M., Hamana K., Hiraishi A. (2001). Proposal of the genus *Sphingomonas sensu stricto* and three new genera, *Sphingobium*, *Novosphingobium* and *Sphingopyxis*, on the basis of phylogenetic and chemotaxonomic analyses. Int. J. Syst. Evol. Microbiol..

[26] Zhang Y., Tay J. (2015). Toxic and inhibitory effects of trichloroethylene aerobic co−metabolism on phenol−grown aerobic granules. J. Hazard. Mater..

[27] Inoue K., Habe H., Yamane H., Omori T., Nojiri H. (2005). Diversity of carbazole−degrading bacteria having the car gene cluster: Isolation of a novel gram−positive carbazole−degrading bacterium. FEMS Microbiol. Lett..

